# Hp-**β**-CD-Voriconazole *In Situ* Gelling System for Ocular Drug Delivery: *In Vitro*, Stability, and Antifungal Activities Assessment

**DOI:** 10.1155/2013/341218

**Published:** 2013-05-09

**Authors:** Pravin Pawar, Heena Kashyap, Sakshi Malhotra, Rakesh Sindhu

**Affiliations:** Chitkara College of Pharmacy, Chitkara University, Chandigarh-Patiala National Highway, Rajpura, Patiala, Punjab 140401, India

## Abstract

The objective of the present study was to design ophthalmic delivery systems based on polymeric carriers that undergo sol-to-gel transition upon change in temperature or in the presence of cations so as to prolong the effect of HP-**β**-CD Voriconazole (VCZ) *in situ* gelling formulations. The *in situ* gelling formulations of Voriconazole were prepared by using pluronic F-127 (PF-127) or with combination of pluronic F-68 (PF-68) and sodium alginate by cold method technique. The prepared formulations were evaluated for their physical appearance, drug content, gelation temperature (*T*_gel_), *in vitro* permeation studies, rheological properties, mucoadhesion studies, antifungal studies, and stability studies. All batches of *in situ* formulations had satisfactory pH ranging from 6.8 to 7.4, drug content between 95% and 100%, showing uniform distribution of drug. As the concentration of each polymeric component was increased, that is, PF-68 and sodium alginate, there was a decrease in *T*_gel_ with increase in viscosity and mucoadhesive strength. The *in vitro* drug release decreased with increase in polymeric concentrations. The stability data concluded that all formulations showed the low degradation and maximum shelf life of 2 years. The antifungal efficiency of the selected formulation against *Candida albicans* and *Asperigillus fumigatus* confirmed that designed formulation has prolonged effect and retained its properties against fungal infection.

## 1. Introduction

In the ophthalmic drug delivery systems, protective barriers of eye lead to low absorption of drug and it leads to poor bioavailability of therapeutic drugs. The *cul*-*de*-*sac* normally holds 7–9 *μ*L of tears but can retain up to approximately 20–30 *μ*L without overflowing. The normal tear flow rate and film thickness are 1 *μ*L/min and 4–9 *μ*m. The normal pH of the tears is ~6.5–7.6. The drainage of instilled solutions (25–50 *μ*L) away from the front of the eye is essentially completed at around 90 sec. Under normal conditions, the eye can accommodate only a very small volume without overflowing. Commercial eye drops have a volume of ~30 *μ*L, which is about the volume of the conjunctival sac in humans; however, after a single blink, only an estimated 10 *μ*L remains [1]. The poor bioavailability and less therapeutic response of convential eye drops occurs mainly due to the gravity induced lacrimal flow and normal tear turnover of the eye. Frequent dosing is usually associated with nonpatient compliance and tear drainage of the administered dose passes via the nasolacrimal duct into the gastrointestinal tract, leading to side effects. Due to this drug loss in front of the eye, very small drug is available to enter the cornea and inner tissue of eye. Its leads to very small corneal contact time (about 1-2 mins) in humans for instilled solution usually less than 10%. Therefore, only small amount of drug actually penetrates the cornea and reaches intraocular surface [2, 3].

An ideal ophthalmic drug delivery must be able to release the drug in sustained manner and remain in the front of eye for prolong period of the time. As a result, various attempts have been made to prolong the contact time of drug on the ocular surface and also to slow down the drug elimination [4], that is, development of viscous gel to prolong the precorneal drug retention [5, 6], microparticle suspension [7], or polymeric solution [8], inserts [9], and collagen shields [10]. However, these dosage forms also comprise some disadvantages such as discomfort especially in elderly patients, loss of device during sleep, or rubbing eye and poor compliance, as well as blurred vision.

The ophthalmic drug delivery based on *in situ gel* can overcome these problems. As *in situ *activated gel forming systems can be administered in drop form and create considerably fewer problems with vision and also provide better sustained properties than drops these *in situ *gelling systems consist of polymers that exhibit sol-to-gel phase transitions due to change in specific physicochemical parameters (pH, temperature, and ionic strength) in the environment, *cul-de-sac* in the case of eye [11]. There are different approaches used for triggering the *in situ* gel formation: physiological stimuli (e.g., temperature induced and pH induced), physical changes in biomaterials (e.g., diffusion of solvent and swelling), and chemical reactions (e.g., enzymatic, chemical, and photoinitiated polymerization).

Polymers such as pluronics (poly(ethylene oxide)-poly(propylene oxide)-poly(ethylene oxide) (PEO-PPOPEO) Triblock), polymer networks of poly(acrylic acid) (PAA), and polyacrylamide (PAAm) or poly(acrylamide-co-butyl methacrylate) are temperature-induced polymers which are liquid at room temperature (20°C–25°C) and undergo gelation when arrive in contact with body fluids (35°C–37°C), due to an increase in temperature [12, 13]. Certain polymers such as PAA (Carbopol, carbomer) or its derivatives, mixtures of poly(methacrylic acid) (PMA) and poly(ethylene glycol) (PEG), show change from sol to gel with change of pH [14, 15]. In presence of various ion such as k^+^, Ca^+2^, Mg^+2^, and Na^+^, certain ion sensitive polysaccharides such as carrageenan, gellan gum (Gelrite), pectin, and sodium alginate undergo phase transition [16, 17]. Poloxamer 407 (PF-127) is a nonionic surfactant composed of poly(ethylene oxide)-b(poly(propylene oxide)-b-poly(ethylene oxide) (PEO-PPO-PEO) showing amphiphilic behavior due to hydrophobic propylene oxide domains and hydrophilic ethylene oxide domains. Pluronic F127 exhibits sol to gel transition at 37°C when used at a higher concentration of (25%–30%) (w/v). By using different series of poloxamers, cross-linking agents, by changing pH and ionic strength gelation, temperature can be adjusted within the range of 33–36°C [18–20].

With most common treatments, amphotericin B and natamycin fungal ulcers tend to have very poor outcomes. Since 1960, no new medication has been approved by the FDA and there has been only a single randomized trial of antifungal therapy for fungal ulcers. There are studies that indicate that the newer triazoles, such as voriconazole, are more effective *in vitro* against filamentous fungi such as *Aspergillus* species, a common cause of fungal keratitis [21]. Voriconazole is a broad spectrum antifungal agent and is commonly used in fungal keratitis and also active against species that are known to be resistant to the other antifungal agents. It is a second-generation synthetic derivative of fluconazole. Voriconazole differs from fluconazole by the addition of a methyl group to the propyl back bone and by the substitution of a triazole moiety with a fluoropyrimidine group, resulting in increased activity of drug. Antifungal potency increased by the substitution of a triazole ring with a pyrimidine moiety, and the addition of a fluorine to this ring structure at the 5 position enhanced *in vivo* efficacy [22].

Voriconazole is a lipophilic drug with a low pH-dependent aqueous solubility (maximum 2.7 mg/mL at pH 1.2). Due to low solubility of voriconazole, it is encapsulated in *β*-cyclodextrin derivative in order to increase the solubility and stability of Voriconazole in aqueous solutions, while maintaining its lipophilicity and high corneal permeability [23, 24]. Hydroxypropyl-*β*-cyclodextrin (HP-*β*-CD), a cyclic oligosaccharide with outer hydrophilic surface and a lipophilic cavity, is capable of forming inclusion complexes with many lipophilic drugs. Solubility enhancement studies of indomethacin conducted using SBE-*β*-CD and HP-*β*-CD have revealed the better potential of HP-*β*-CD as a solubility enhancing agent [25]. Ciprofloxacin ophthalmic formulations prepared using HP-*β*-CD demonstrated better stability and biological activity than the ophthalmic solution without HP-*β*-CD [26]. Voriconazole has a good penetration through the cornea into the aqueous humour and does not affect intraocular safety when administered topically [27].

The objective of present study was to develop and evaluate a temperature triggered *in situ* ophthalmic gel system for voriconazole. 

## 2. Experimental

### 2.1. Materials

Voriconazole and hydroxypropyl-*β*-cyclodextrin were received as gift samples from Matrix Laboratories, Hyderabad (India), pluronics (F-127), pluronics (F-68), sodium alginate, and mucin from porcine stomach type II were purchased from Sigma-Aldrich Pvt. Ltd., (India), and Hi Media Sabouraud Dextrose Agar was obtained from Deep Scientific Laboratories, Chandigarh, (India). All other chemicals used were of analytical reagent grade. Freeze-dried strains *Candida albicans* (MTCC 227) and* Aspergillus fumigatus* (MTCC 2544) were obtained from MTCC, IMTECH Chandigarh, India; Fresh whole eyeballs of goat were obtained from local butcher's shop (Zirakpur, Punjab, India) within one hour of slaughtering of animal.

### 2.2. Preparation of Voriconazole *In Situ *Gels

*In situ *gelling liquids were prepared using different concentrations of pluronic F-68 and sodium alginate with fixed concentration of pluronic F-127. Voriconazole (0.15 w/v) was weighed separately and dissolved in the distilled water with (1.5%w/v) HP-*β*-CD. Sodium alginate solutions of different concentrations (0.5%, 1%, and 1.5%) were prepared by dispersing the required amount in distilled water with continuous stirring until completely dissolved. The voriconazole solution was added to the alginate solution under constant stirring until uniform, clear solution was obtained. Further, to this mixture pluronic F-127 (15% w/v) and different concentrations of pluronic F-68 (14%, 15%, and 16%) were added. Benzalkonium chloride (0.01% w/v) was added as a preservative to the previous solutions. Sufficient amount of sodium chloride was added to the mixture to maintain the isotonicity. Finally, the volume was adjusted with distilled water up to 100 mL. Partially the dissolved pluronic solutions were stored overnight in a refrigerator at 4°C for hydration and stirred periodically until clear homogenous solutions were obtained. Nine batches of formulation were prepared by using different concentrations of sodium alginate and PF-68 as shown in Table 2. 

### 2.3. Physicochemical Evaluation of *In Situ *Gelling Formulations

#### 2.3.1. Measurement of Gelation Temperature

At room temperature, ten milliliters of cold sample solution (pluronic containing formula) were put into a beaker (25 mL) and placed in a low temperature water bath. A thermometer was immersed into the sample solution for constant monitoring. The solution was heated with stirring at 200 rpm using a magnetic bar (9 × 25 mm). The temperature at which the magnetic bar stopped moving due to gelation was reported as the gelation temperature (*T*_gel_). Each sample was measured in triplicate [20, 28].

#### 2.3.2. Drug Content Uniformity

Drug content of Voriconazole *in situ* gelling formulations was determined by accurately dissolving (1 mL) weighed quantity of formulation in 100 mL simulated tears fluid. The formulation was shaken for 2-3 min to completely dissolve, until it gives a clear gel solution. The solution was filtered through Millipore membrane filter (0.45 *µ*m) and drug content was analyzed by UV-Vis Spectrophotometry at 260 nm. The experiments were done in triplicate and the mean ± SD was reported [29].

#### 2.3.3. Rheological Studies

It is the important factor to determine the residence time of drug in the eye by considering the viscosity of the instilled formulation. The prepared solutions were allowed to gel at physiological temperature and then the viscosity determination was carried out by using Brookfield viscometer (Brookfield DV+Pro, Brookfield Engineering Laboratories, Middleboro, MA, USA). By plotting graph of shear rate versus shear stress, the flow pattern was checked.

#### 2.3.4. Bioadhesion Strength

To quantify mucin-polymer mucoadhesive strength of gel formulation, a simple viscometric method was used [30]. Viscosities of 15% (w/v) porcine gastric mucin dispersions in STF were measured with a Brookfield viscometer in the absence (*ηm*) or presence (*ηt*) of different formulations at a shear rate of a shear rate of 100 rpm at 37°C. For homogenous distribution throughout the sample, viscometric measurements were performed after exactly 3 min of applying the shear force. Viscosity components of mucoadhesion (*ηb*) were calculated from the equation *ηt* = *ηm* + *ηp* + *ηb*, where *ηp* is the viscosity of corresponding pure polymer solution. The force of mucoadhesion (*F*) was calculated from the equation  *F* = *ηb* · *σ*, where *σ* is the rate of shear/sec. 

#### 2.3.5. *In Vitro* Drug Permeation

The *in vitro *drug permeation studies were carried out by putting the *in situ* gelling formulation on Millipore membrane filter (0.15 mm) between the donor and receptor compartments of an all-glass modified Franz diffusion cell. To simulate the corneal epithelial barrier, the Millipore membrane filter was used, as isolated cornea will not remain viable beyond 4 hr. The receptor compartment of an all-glass modified Franz diffusion cell was filled with 10 mL freshly prepared simulated tear fluid (pH 7.0), and all air bubbles were expelled from the compartment. An aliquot (1 mL) of test solution was placed on the Millipore membrane filter, and the opening of the donor cell was sealed with a glass cover slip. The receptor fluid was kept at 37 ± 0.5°C with constant stirring using a Teflon-coated magnetic stir bead. Permeation study was continued for 10 hr, and samples were withdrawn from receptor and analyzed for Voriconazole content by measuring absorbance at 260 nm in a spectrophotometer (UV-Vis Spectrophotometer 2701 A Systronics, Mumbai, India).

Drug permeation experiments were also carried out using freshly excised goat cornea. Goat whole eyeballs were transported from the local butcher shop to the laboratory in cold (4°C) normal saline within 1 hr of slaughtering of the animal. The cornea was carefully excised along with 2 to 4 mm of surrounding scleral tissue and was washed with cold normal saline till the washing was free from proteins. Freshly excised cornea was fixed between clamped donor and receptor compartments in such a way that its epithelial surface faced the donor compartment. For the analysis of Voriconazole withdrawn from receptor compartment, the same procedure was adopted as mentioned earlier. Results were expressed as cumulative percentage of drug released versus time.

#### 2.3.6. Release Kinetics Study

To study the drug release kinetics, data obtained from *in vitro *permeation studies were fitted in various kinetic models: zero order as the cumulative percent of drug permeated versus time, first order as the log cumulative percentage of drug remaining versus time, and Higuchi's model as the cumulative percent drug permeated versus square root of time. The release mechanism of voriconazole from *in situ* gel was determined by fitting the data into the Korsmeyer-Peppas model as the log cumulative percentage of drug released versus log time, and the exponent “*n*” was calculated from the slope of the straight line. If *n* < 0.45, then the diffusion mechanism is Fickian; if 0.5 < *n* < 0.8, the non-Fickian and *n* > 1 show super case II transport. The drug permeation data was plotted according to zero order, first-order kinetics, Higuchi equation, and Korsmeyer-Peppas equation [31].

#### 2.3.7. Corneal Hydration (HL%)

Wet corneal weight (*W*_*a*_) was noted after removal of cornea from donor compartment after experiment. Each corneal sample soaked in methanol (1 mL) and dried overnight at 90°C and reweighed (*W*_*b*_). The percentage corneal hydration level (HL %) was calculated by the formula corneal  hydration = [1 − (*W*_*a*_/*W*_*b*_  )]∗100 [32].

#### 2.3.8. Antifungal Studies

The antifungal efficiency and prolonged effect of selected sustained release *in situ* gel of voriconazole formulations were carried out on *Candida albicans*  and  *Asperigillus fumigatus *species. The nutrient agar medium was prepared by dissolving saboured dextrose in hot distilled water and media was autoclaved at 121°C for 15 min. By using diffusion method test organisms were previously seeded (10 CFU/mL) in the nutrient agar medium [33]. The aliquot test samples were poured into petri dish containing nutrient agar medium using micropipette. The plates were left for 30 min and then incubated at 25°C for 24 hr. The diameters of zone of inhibition for *Candida albicans* and *Aspergillus fumigatus* were measured after 24 hr and 120 hr, respectively.

#### 2.3.9. Stability of *In Situ *Gel

Stability studies were carried out on *in situ* gelling formulations according to ICH guidelines [34]. All formulations were stored in closed amber glass bottles and placed at humidity chamber with a relative humidity of 75 ± 5% and temperatures of 40 ± 2°C or at room temperature. Samples were withdrawn at time 0, 3 weeks, 6 weeks, 3 months, and 6 months and analyzed for drug concentration. The formulations were evaluated at periodic intervals for pH, clarity, and drug content. The degradation rate constant was determined from the plot of logarithm of the remaining drug versus time. 

### 2.4. Statistical Analysis

All values presented in the study are average of triplicate experiments for the same time points. Differences in viscosities and *in vitro* permeability profile of voriconazole *in situ *gel under different conditions were tested statistically using one-way analysis of variance (ANOVA) followed by Dunnett's test at different level of significance. (^†^Statistically significant difference at *P* < 0.05; ^††^statistically significant difference at *P* < 0.01; ^†††^statistically significant difference at *P* < 0.001 from control.)

## 3. Results and Discussion

Pluronic F127 became one of the most extensively investigated temperature-responsive materials due to its unique thermoreversible gelation properties, but the phase transition temperature strongly depended on pluronic F127 concentration [35]. Pluronic F68 is incorporated into pluronic F127 in order to modulate the phase transisition temperature for ophthalmic drug delivery system [36]. Sodium alginate is a natural hydrophilic polysaccharide containing two types of monomers, b-d-mannuronic acid (M) and a-l-guluronic acid (G). Alginate is not easily eroded by tear fluid as it transforms into stable gel upon exposure to divalent cations and it has also mucoadhesive property [11, 37].

Pluronic 127 (15%, w/v) was selected as the basis of formulation because below this concentration it loses its sol-gel transition properties and it is used in combination with pluronics 68 and sodium alginate in different concentrations. Sodium alginate was combined in formulation for the additive effect of mucoadhesive property.

### 3.1. Clarity, Drug Content, and pH

The physicochemical properties of the Voriconazole formulations are shown in Table 1. The drug content, clarity, and pH of the formulations were found to be satisfactory and the formulations were liquid at both room temperature and refrigerated temperature conditions. For ophthalmic delivery, clarity of formulation is the main concern because acceptability of formulation is based on it. All gels of Voriconazole formulation batches were observed clear and transparent.

All the formulations should have satisfactory pH ranging from 6.8 to 7.4, which is acceptable for ocular delivery. Drug content values ranging from 91.4 ± 0.51 to 99.01 ± 2.79% were showing uniform distribution of drug.

### 3.2. Gelation Temperature (*T*_gel_)

*T*_gel_ is the temperature at which the liquid phase makes a transition to gel. The basic prerequisite for *in situ *gelling system is that the gel formulation should be a free flowing liquid at room temperature for ease to be administrated at site of application in eye where it becomes gel as in nature at physiological temperature of human eye, that is, 37°C [38].

The gelation temperature (*T*_gel_) for the the formulations was found in between range of 24.36 ± 0.41 to 37.33 ± 0.73 (Table 1). The minimum gelation temperature was observed for batch VG9, that is, 24.36 ± 0.41. This might be effect of higher concentration of PF-68 (16%) and with combination of sodium alginate (1.5%). The result also suggests that the increased concentration of P-68 in *in situ *gel decreases the gelation temperature of formulation. The earlier literature reported that the addition of pluronic PF-68 in formulation can lead to micellar entanglement and changing the PEO/PPO ratio [39]. In micellation, on one hand, the in aqueous solution forms an aggregate with the hydrophilic head, and on the other, aqueous solvent is sequestered with the hydrophobic single-tail regions in the micelle centre. The formation of micelles might increase the viscosity of vehicles and end up in forming a gel.

Sodium alginate is also responsible for decreasing gelation temperature. As the concentration of sodium alginate increases, that is, 1.5%  *T*_gel_ decreases as shown in batches (VG3, VG6, VG9), it reduced due to more entangled nature of the polymeric networks [40].

### 3.3. Rheological Viscosity

**Figure 1:**
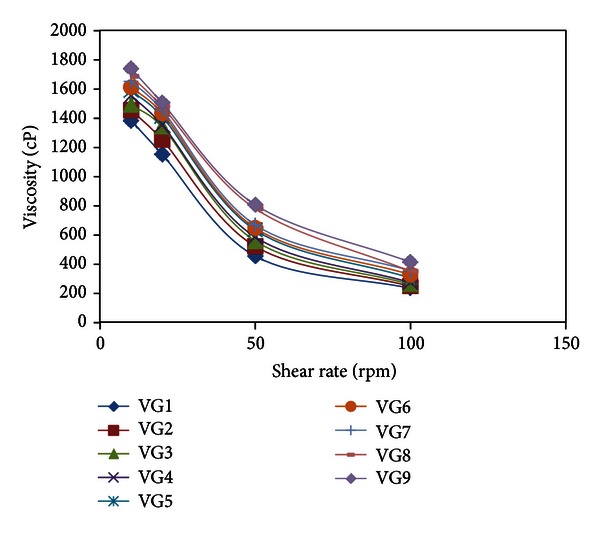
Viscosity profile of all Voriconazole *in situ* gels at different shear rates (rpm).

The viscosity values of all formulations were shown in Table 2. The result suggests that all the formulations provide pseudoplastic behavior as shown in Figure 1. Among all the formulations, VG9 formulation provides maximum viscosity and statistically significant (*P* < 0.01) value, that is, 2698 ± 3.79 cps at 100 rpm (37°C). Viscosity is an important factor to determine the residence time of drug in the eye. In ocular drug delivery system, the ophthalmic products should not disturb the pseudoplastic character of precorneal tear film. The ocular shear rate is about 0.03 s^−1^ during interblinking periods and 4250–28500 s^−1^ during blinking. So, the viscoelastic fluids having high viscosity under low shear rates and low viscosity under high shear rates called as pseudo plastic fluid are often preferred [41]. All the formulations exhibited pseudo plastic behavior; that is, with increase in shear rate, a decrease in viscosity was observed. This might be due to that gel formation (*T*_gel_) decreases as concentration of pluronics (P-68) and sodium alginate increases as a result of micellar enlargement and packing. This result also contributes that due to these micelle entanglements, they cannot separate easily from each other, which leads to the high viscosity of gels containing high concentrations of pluronics (P-68) and sodium alginate.

### 3.4. Bioadhesive Strength

The force of bioadhesion is an important and crucial physicochemical parameter for *in situ *forming ophthalmic gels since it prevents the formulation from rapid drainage, and hence, prolongs its residence time. The values of all bioadhesive forces were depicted in Table 2. The force of bioadhesion of formulation VG9 was observed maximum (28.28 dynes/cm^2^), that is, statistically significant (*P* < 0.01) compared with other formulation. The rest of formulation provides the bioadhesion force in between range of 17.24 to 27.59 dynes/cm^2^. The Voriconazole *in situ* gel formulation VG9 exhibits maximum bioadhesion force due to combination effect of sodium alginate and PF-68. As the viscosity of VG9 was maximum, that is, (2698 ± 3.79 cps), that might contribute to higher force of bioadhesion as compared to other formulations. Increasing the mucoadhesive polymer, sodium alginate concentration in the formulation significantly increased the mucoadhesive force of the formulation. Addition of PF-68 enhanced the bioadhesive force, since the pluronic with a hydrophilic oxide group could bind to oligosaccharide chains. The higher the concentration of PF-68, the greater the bioadhesive force of pluronic gel.

### 3.5. *In Vitro* Drug Permeation

The *in vitro* permeation studies of Voriconazole *in situ *gel were carried out through the Millipore membrane filter paper (0.15 *µ*m) and freshly excised goat cornea, clamped between donor and receptor compartments of all glass modified Franz diffusion cell. Formulation VG3 containing higher concentration of sodium alginate with respect to formulation VG1 and VG2 showed lesser amount of drug through Millipore membrane filter at the end of 1 hr and same release pattern was obtained at 10 hr (Table 3).

**Figure 2:**
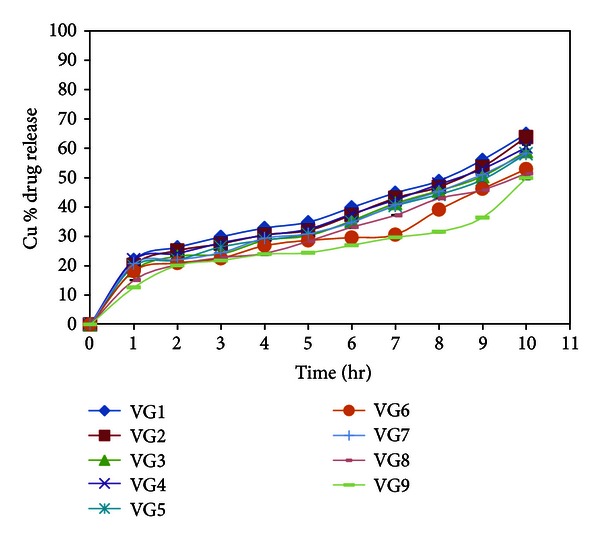
*In vitro* permeation profile of Voriconazole from *in situ* gelling systems through Millipore membrane filter.

The formulation VG6 containing 15% w/v of PF-68 and a comparitively higher concentration (1.5%) of sodium alginate, showed a cumulative release of 53% at the end of 10 hr which indicated that VG6 provided a sustained release. The results are shown in Figure 2 showing statistically significant values (*P* < 0.01), which indicates that the *in vitro* permeation from *in situ* gel of Voriconazole (VG1 to VG9) was sustained for 10 hr. The drug permeated from the formulation VG9 showed a lesser cumulative percantage release in comparison to the other formulations and hence demonstrated the maximum sustained release. The effect of PF-68 on the release rate of VCZ from the pluronic-based *in situ* gelling formulations showed little effect on the release rate of VCZ from *in situ* gelling formulations. The addition of PF-68 (16%) resulted in a decrease in the drug release rate compared to PF-68 (14%) and PF-68 (15%). The results indicate that as the concentration of PF-68 and sodium alginate increases, *T*_gel_ decreases due to micellar entanglement, leading to higher viscosity of the gel which functioned as an increasingly resistant barrier to drug release. Due to increase in the number and size of micelles within the gel structure, it leads to the enhanced resistance resulting in a more entangled system and more rigid gel and also attributed to the increase in viscosity.

**Figure 3:**
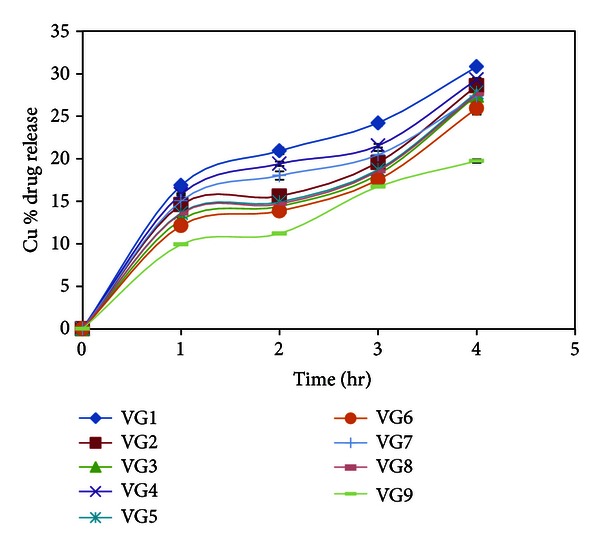
*In vitro* permeation profile of Voriconazole from *in situ* gelling systems through freshly excised goat cornea.

The *in vitro* drug permeation studies of *in situ *gel formulations of voriconazole through excised goat corneas are shown in Table 4 and Figure 3. To mimic real life conditions, excised goat corneas were used for permeation studies and the experiment was conducted for 4 hr considering cornea viability, and the drug permeation from *in situ* gels ranged between 30.82 and 19.74%, which was less than the permeation observed with the Millipore membrane filter in 4 hr. In goat corneas permeation studies, the formulation VG9 showed 19.74 ± 0.21% release at the end of 4 hour which is less as compared to other formulations.

The rank order of drug release was VG1 > VG2 > VG4 > VG3 > VG7 > VG5 > VG6 > VG8 > VG9 for Millipore membrane filter as well as in goat cornea. Cornea (made of epithelium (lipophilic), stroma (hydrophilic), and endothelium (less lipophilic than epithelium)) acts as a lipophilic-hydrophilic barrier and the drug will have to partition through the barrier for corneal penetration while Millipore membrane filter acts as a mechanical barrier to drug diffusion. Accordingly, permeation through the cornea would be lower compared to that across the Millipore filter paper.

### 3.6. Release Kinetics Study

Release kinetic models are shown in Table 4. The table indicates that the correlation coefficient of release data fits more into the Higuchi model for most number of cases than any other available models (using Millipore membrane and goat cornea as permeation medium). The release profiles of *in situ* gelling formulations were treated with the Korsmeyer-Peppas equation, and slope values *n* > 0.89 were indicating anamolous drug release involving a combination of both Fickian and non-Fickian diffusions through the Millipore membrane filter and excised goat cornea. 

### 3.7. Corneal Hydration

The corneal hydration level of normal mammalian cornea is between 75% and 80% [42]. The drug concentration, pH, and addition of preservatives and/or polymers in Voriconazole *in situ* gelling formulations did not show any corneal damage as the corneal hydration value for all corneas remained in the normal range of 75% to 80% (Table 3). 

### 3.8. Microbiological Assay

The antifungal efficiency of the selected controlled release voriconazole formulation VG9 was evaluated against organisms including *Candida albicans *and *Aspergillus fumigatus.* The mean diameters of zone of inhibition with *Candida albicans* and *Aspergillus fumigatus *are depicted in Table 5. For the preparation of Control 2, the same set of procedures was followed as employed in case of the formulation VG9, except for the use of HP-*β*-CD. This was done to check the antifungal potential of voriconazole alone in absence of HP-*β*-CD. The microbiological assay studies conducted using agar diffusion method indicated that HP-*β*-CD based Voriconazole *in situ *gel formulation VG9 inhibited the growth of *Candida albicans* and *Aspergillus fumigatus, *while the control solutions of VG9 formulation without drug (Control 1) and VG9 without HP-*β*-CD (Control 2) did not inhibit the fungal growth. The inhibition zones were evaluated after 24 hours, and reduction in the growth of microorganisms was clearly observed. The zone of inhibition increased significantly (*P* < 0.01) as the amount of Voriconazole diffused from the *in situ* gel was increased.

### 3.9. Stability Studies

**Figure 4:**
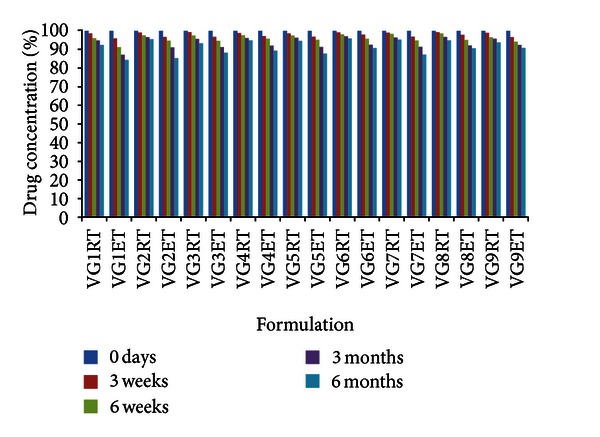
Stability of Voriconazole *in situ *gels under accelerated temperature and room temperature. Mean ± SD (*n* = 3). RT: room temperature (30°C); ET: elevated temperature (40°C).

Finally, accelerated stability studies at elevated temperature and humidity revealed no significant changes in pH and clarity of *in situ* gelling formulations. The Voriconazole concentrations in all formulations at accelerated and room temperature are shown in Figure 4. The degradation rate constants (*k*cal) and shelf life (*t*90) were found to range between 2.61 and 2.12 days^−1^ and 889–694 days. The stability studies concluded that all formulations showed the lowest degradation and maximum stability of 2 years.

## 4. Conclusions

In conclusion, we suggest that the *in situ* gelling formulations of HP-*β*-CD based Voriconazole can be a promising vehicle for topical ocular administration of antifungal against *Candida albicans *and *Aspergillus fumigatus*. Its application could reduce the necessity for repeated drug administration at frequent intervals due to the sustained release of the formulation, thereby potentially lowering corneal toxicity and increasing patient compliance.

## Figures and Tables

**Table 1:** Physiochemical characterization of *in situ *gels of Voriconazole, mean ± SD; *n* = 3.

Formulations	Concentration		Gelation temperature (°C)	Drug content %	pH
	*PF-68	*Na alginate			
VG1	14	0.5	37.33 ± 0.73	96.98 ± 1.34	6.8
VG2	14	1	36.50 ± 0.35	96.34 ± 2.00	6.9
VG3	14	1.5	35.70 ± 0.36	93.33 ± 1.26	7.0
VG4	15	0.5	33.80 ± 0.40	92.34 ± 2.15	6.9
VG5	15	1	32.60 ± 0.30	94.91 ± 0.372	7.1
VG6	15	1.5	30.83 ± 0.65	99.01 ± 2.79	7.4
VG7	16	0.5	29.63 ± 0.51	97.09 ± 1.90	6.9
VG8	16	1	28.36 ± 0.41	92.34 ± 0.66	6.8
VG9	16	1.5	24.36 ± 0.41	91.41 ± 0.51	7.2

*PF 68—Pluronic 68 or Poloxamer 118.

*Na alginate—sodium alginate.

**Table 2:** Effect of the addition of different concentrations of sodium alginate and P-68 to voriconazole *in situ* gelling formulation on viscosity of gel, bioadhesion component, and force of bioadhesion.

Formulations	Viscosity of gel at 100 rpm (cP)	Viscosity with bioadhesive component at 100 rpm (cP)	Force of bioadhesion (dyne/cm^2^)
VG1	235.7 ± 6.66	1857 ± 6.42	17.24
VG2	249.3 ± 4.93^††^	1963 ± 6.55^††^	18.78
VG3	262.3 ± 7.02^††^	2047 ± 4.16^††^	19.96
VG4	274.3 ± 5.03^††^	2186 ± 2.08^††^	22.08
VG5	306.3 ± 6.11^††^	2236 ± 4.58^††^	22.38
VG6	329.3 ± 10.5^††^	2345 ± 3.21^††^	23.82
VG7	355.7 ± 8.50^††^	2478 ± 4.01^††^	25.59
VG8	351.7 ± 28.3^††^	2576 ± 3.11^††^	27.59
VG9	414.3 ± 10.0^††^	2698 ± 3.79^††^	28.28

Values are mean ± SE of 3 gel viscosities in each group.

^†^Statistically significant difference at *P* < 0.05.

^††^Statistically significant difference at *P* < 0.01.

^†††^Statistically significant difference at *P* < 0.001 from control (VGI containing 15% PF-127, 14% PF-68, 0.5% sodium alginate) as determined by one-way ANOVA followed by Dunnett's test.

**Table 3:** *In vitro* permeation of Voriconazole from *in situ* gels through millipore membrane filter and freshly excised goat cornea.

% Drug permeation*					
Formulations	Millipore paper		Goat cornea		Corneal hydration (%)
	*t*_1_	*t*_10_	*t*_1_	*t*_4_	
VG1	22.01 ± 0.21	64.98 ± 0.65	16.89 ± 0.27	30.82 ± 0.27	77.03 ± 1.9
VG2	20.27 ± 0.23^††^	63.82 ± 1.07^††^	14.61 ± 0.15^††^	28.58 ± 0.12^††^	79.80 ± 0.6
VG3	18.47 ± 0.10^††^	59.01 ± 0.35^††^	12.82 ± 0.12^††^	27.46 ± 0.19^††^	78.71 ± 2.3
VG4	21.98 ± 0.19^††^	60.32 ± 0.80^††^	15.84 ± 0.08^††^	29.31 ± 0.26^††^	75.69 ± 0.5
VG5	19.89 ± 0.08^††^	58.13 ± 0.27^††^	13.64 ± 0.41^††^	27.80 ± 0.46^††^	74.90 ± 1.7
VG6	18.24 ± 0.13^††^	52.96 ± 0.38^††^	12.14 ± 0.52^††^	25.94 ± 0.72^††^	76.33 ± 3.1
VG7	20.69 ± 0.21^††^	58.30 ± 0.39^††^	15.04 ± 0.43^††^	27.37 ± 1.70^††^	77.05 ± 0.9
VG8	15.10 ± 0.12^††^	51.47 ± 0.47^††^	13.57 ± 0.46^††^	27.60 ± 0.47^††^	79.02 ± 1.7
VG9	12.64 ± 0.18^††^	49.84 ± 0.49^††^	9.93 ± 0.10^††^	19.74 ± 0.21^††^	75.90 ± 0.6

*Mean ± SD, *n* = 3.

*t*_1_—cumulative percent drug after 1 hr; *t*_10_—cumulative percent drug after 10 hr; *t*_4_—cumulative percent drug after 4 hr.

^†^Statistically significant difference at *P* < 0.05.

^††^Statistically significant difference at *P* < 0.01.

^†††^Statistically significant difference at *P* < 0.001 from control (VGI containing 15% PF-127, 14% PF-68, 0.5% sodium alginate) as determined by one-way ANOVA followed by Dunnett's test.

**Table 4:** Kinetic profiles of *in vitro* drug release from *in situ* gels through Millipore membrane filter and freshly excised goat cornea.

Formulations	(*R* ^2^)								
	Zero order		1st order		Higuchi		Korsmeyer-Peppas		
	Millipore goat	Membrane cornea	Millipore goat	Membrane cornea	Millipore goat	Membrane cornea	Millipore goat	Membrane cornea	Mechanism of drug release
VG1	0.9286	0.8887	0.9326	0.9176	0.9505	0.9866	0.9126	0.9474	Fickian
VG2	0.9320	0.9006	0.9227	0.9128	0.9362	0.9414	0.8981	0.7705	Non-Fickian
VG3	0.9461	0.9207	0.9227	0.9269	0.9452	0.9346	0.8981	0.8073	Non-Fickian
VG4	0.9298	0.8840	0.9438	0.9077	0.9506	0.9738	0.9337	0.8922	Fickian
VG5	0.9338	0.9102	0.9402	0.9195	0.9478	0.9388	0.9015	0.7878	Fickian
VG6	0.9011	0.9250	0.8985	0.9325	0.9183	0.9428	0.9077	0.8304	Fickian
VG7	0.9376	0.8828	0.9435	0.9059	0.9560	0.9755	0.896	0.8947	Fickian
VG8	0.9587	0.9089	0.9535	0.9179	0.9620	0.9366	0.9451	0.7789	Non-Fickian
VG9	0.8812	0.9311	0.8632	0.9440	0.9034	0.9750	0.9023	0.890	Non-Fickian

**Table 5:** A comparative study of anti-fungal activity of voriconazole *in situ *gel against *Candida albicans *and *Aspergillus fumigatus. *

S.NO.	Solution	Mean of diameter of zone of inhibition (mm) ± SE	Range of zone size (mm)	Coefficient of variance (%)
*Candida albicans *	Test	32.33 ± 0.16	32.11–32.48	0.49
	Control 1	10.04 ± 0.03	10.01–10.12	0.30
	Control 2	12.05 ± 0.04	12.01–12.09	0.04
*Aspergillus fumigates *	Test	68.19 ± 0.56	68.01–69.01	0.81
	Control 1	10.02 ± 0.01	10.01–10.14	0.15
	Control 2	12.07 ± 0.02	12.05–12.09	0.17

*Test—VG9 formulation.

*Control 1—VG9 formulation without drug.

*Control 2—VG9 formulation without HP-*β*-CD.
